# Study on ZrSi_2_ as a Candidate Material for Extreme Ultraviolet Pellicles

**DOI:** 10.3390/membranes13080731

**Published:** 2023-08-14

**Authors:** Seong Ju Wi, Won Jin Kim, Haneul Kim, Dongmin Jeong, Dong Gi Lee, Jaehyuck Choi, Sang Jin Cho, Lan Yu, Jinho Ahn

**Affiliations:** 1 Division of Materials Science and Engineering, Hanyang University, Seoul 04763, Republic of Korea; wsj1992@hanyang.ac.kr (S.J.W.); kwj7487@gmail.com (W.J.K.); skylife103@hanyang.ac.kr (H.K.); dmjeong93@gmail.com (D.J.); ehdrl1987@naver.com (D.G.L.); 2 EUV-IUCC (Industry University Cooperation Center), Hanyang University, Seoul 04763, Republic of Korea; 3 R&D Center, FINE SEMITECH CORP, Hwaseong-si 18487, Republic of Korea; choijh@fstc.co.kr (J.C.); sj.cho@fstc.co.kr (S.J.C.); lyu@fstc.co.kr (L.Y.)

**Keywords:** EUV pellicle, zirconium silicide (ZrSi_2_), EUV transmittance, EUV reflectivity, emissivity, ultimate tensile strength

## Abstract

An extreme ultraviolet (EUV) pellicle is an ultrathin membrane at a stand-off distance from the reticle surface that protects the EUV mask from contamination during the exposure process. EUV pellicles must exhibit high EUV transmittance, low EUV reflectivity, and superior thermomechanical durability that can withstand the gradually increasing EUV source power. This study proposes an optimal range of optical constants to satisfy the EUV pellicle requirements based on the optical simulation results. Based on this, zirconium disilicide (ZrSi_2_), which is expected to satisfy the optical and thermomechanical requirements, was selected as the EUV pellicle candidate material. An EUV pellicle composite comprising a ZrSi_2_ thin film deposited via co-sputtering was fabricated, and its thermal, optical, and mechanical properties were evaluated. The emissivity increased with an increase in the thickness of the ZrSi_2_ thin film. The measured EUV transmittance (92.7%) and reflectivity (0.033%) of the fabricated pellicle satisfied the EUV pellicle requirements. The ultimate tensile strength of the pellicle was 3.5 GPa. Thus, the applicability of the ZrSi_2_ thin film as an EUV pellicle material was verified.

## 1. Introduction

Extreme ultraviolet (EUV) lithography has been applied in high-volume manufacturing of advanced semiconductor devices at a sub-7-nm technology node [1,2,3]. An EUV pellicle is a freestanding membrane that protects the EUV mask from the external defects generated inside the EUV scanner [4,5]. The EUV pellicle must exhibit an EUV transmittance higher than 90% and an EUV reflectivity lower than 0.04% to minimize throughput and yield losses. In addition, it must be mechanically and chemically stable inside the EUV scanner and exhibit adequate thermal durability to withstand a high-power EUV source [5,6,7,8,9]. However, the thickness of the EUV pellicle must be in the order of several tens of nanometers to limit the high absorption of EUV light. Various materials are being examined as EUV pellicle candidates to simultaneously achieve satisfactory thermal, mechanical, and chemical properties at a limited thickness. However, EUV pellicle materials that have been studied in the past do not satisfy these requirements. Si, which possesses the highest EUV transmittance, has limitations in terms of thermomechanical durability at high temperatures. To compensate for this, Ru has been investigated as a thermal emission layer; however, its optical characteristics are limited due to a EUV reflectivity higher than 0.04% [6].

Zr-Si intermetallic compounds are anticipated to exhibit higher EUV transmittance and lower EUV reflectivity than other materials at a wavelength of 13.5 nm because of their low extinction coefficient (k) and a refractive index (n) close to 1 [10,11]. Since zirconium disilicide (ZrSi_2_) is used as a spectral purity filter inside the EUV scanner, it is expected to have superior thermomechanical durability [11,12,13,14]. Moreover, ZrSi_2_ has a high Young’s modulus and high compressive yield strength at high temperatures [15]. However, ZrSi_2_ has not been previously examined as a candidate for EUV pellicle materials.

In this study, we examined the optical constant conditions for an EUV pellicle to achieve superior optical performance using an optical simulation tool. Based on the results of the simulation, ZrSi_2_ was proposed as an EUV pellicle candidate considering its optical constant and thermomechanical properties. An EUV pellicle composite containing the ZrSi_2_ thin film was fabricated. Its optical, thermal, and mechanical properties were evaluated to verify the potential application of ZrSi_2_ as an EUV pellicle material.

## 2. Material Selection for Application as an EUV Pellicle

The EUV transmittance and reflectivity were calculated using the PROLITH 2022a rigorous coupled-wave analysis simulation tool to identify the optimal conditions for optical constants.

**Figure 1:**
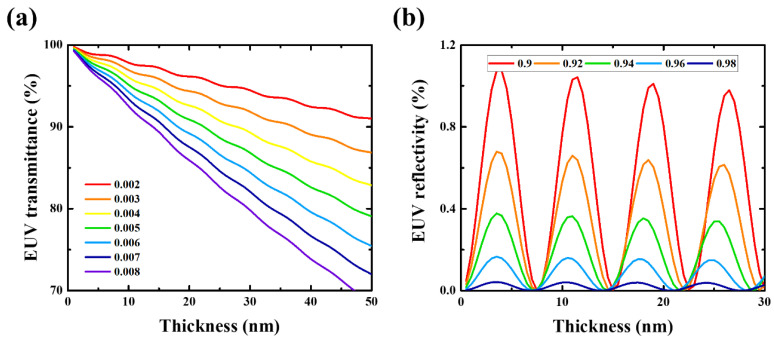
Simulation results of (**a**) EUV transmittance according to the extinction coefficient and (**b**) EUV reflectivity according to the refractive index of the membrane at various thicknesses.

Figure 1a shows the simulation results of EUV transmittance with respect to the extinction coefficient at a refractive index of 0.94. The results confirmed that a higher extinction coefficient results in a sharper decrease in the EUV transmittance when the thickness of the thin film is increased. When the extinction coefficient is higher than 0.006, an EUV transmittance greater than 90% can be achieved only if the thickness of the film is 20 nm or less. However, fabrication of a free-standing membrane with a thickness of 20 nm or less is difficult. Figure 1b shows the simulation results of the EUV reflectivity according to the refractive index for an extinction coefficient of 0.005. A smaller thickness margin that satisfies the EUV reflectivity requirement was observed as the refractive index decreased. At an extremely small thickness margin, obtaining a thin film with a thickness that satisfies the EUV reflectivity requirements becomes difficult. Therefore, the refractive index must be greater than 0.94.

**Figure 2:**
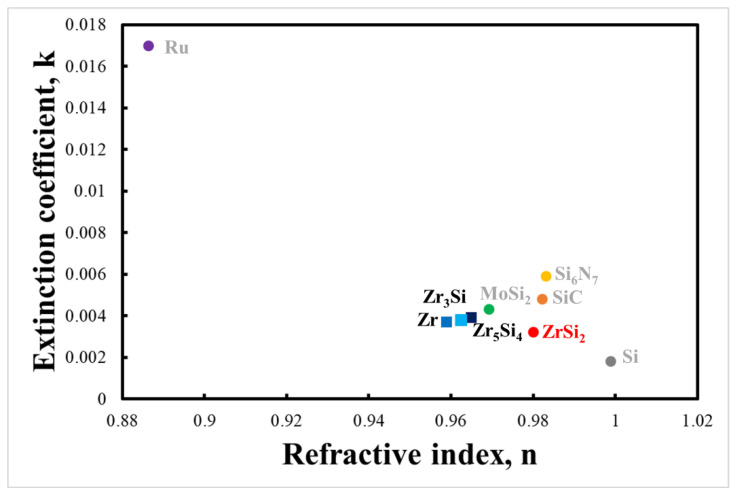
Optical constants map at a wavelength of 13.5 nm for EUV pellicle candidates.

Figure 2 shows the refractive indices and extinction coefficients of various materials at a wavelength of 13.5 nm [16]. Zr and Zr-Si intermetallic compounds exhibited lower extinction coefficients than other EUV pellicle candidates [17]. Among Zr-Si intermetallic compounds, ZrSi_2_ has the lowest extinction coefficient and the highest refractive index, and, hence, ZrSi_2_ is expected to exhibit excellent optical properties. The EUV transmittance and reflectivity were simulated as a function of the composition ratio and thickness of the Zr-Si intermetallic compounds, and an optimal composition was determined.

**Figure 3:**
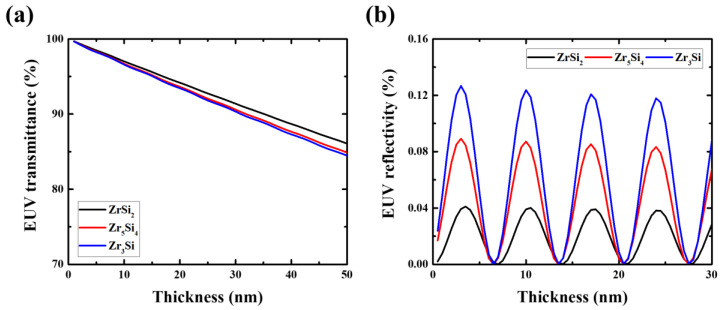
Simulation results of (**a**) EUV transmittance and (**b**) EUV reflectivity according to the composition ratio of zirconium silicide (ZrSi_x_).

Figure 3 shows the results of EUV transmittance and reflectivity simulations. The ZrSi_2_ thin film exhibited the highest EUV transmittance. In addition, the ZrSi_2_ thin film satisfied the EUV reflectivity requirement of 0.04% or less for most thicknesses. Therefore, ZrSi_2_, which is expected the most promising material for obtaining optical properties, was selected as the EUV pellicle candidate.

## 3. Experimental Details

**Figure 4:**
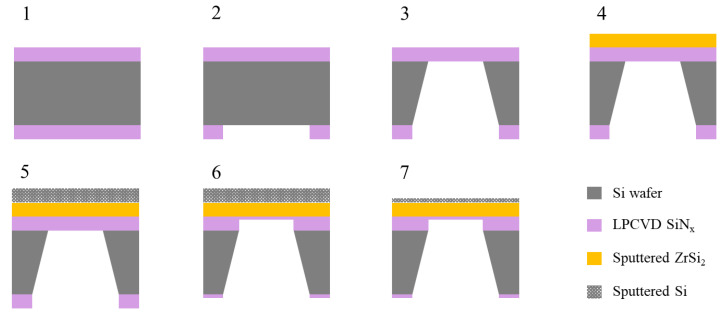
Fabrication process of EUV pellicle including the ZrSi_2_ thin film.

**Figure 5:**
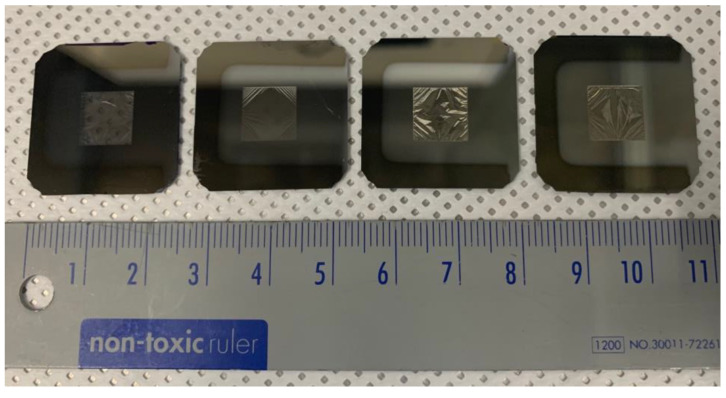
Fabricated ZrSi_2_/SiN_x_ pellicle composite.

Figure 4 shows a schematic of the EUV pellicle fabrication process, including the ZrSi_2_ thin film used in this study. A 40-nm-thick silicon nitride (SiN_x_) thin film was deposited on both sides of a (100) p-type silicon wafer via a low-pressure chemical vapor deposition process at 800 °C using ammonia (NH_3_) and dichlorosilane (DCS, SiH_2_Cl_2_) gas. A photoresist was coated on the back, and the backside window was obtained via photolithography. Thereafter, the membrane area was patterned via reactive ion etching using CF_4_, CHF_3_, and O_2_ as reactant gases and Ar as the carrier gas. A free-standing membrane was fabricated by etching a silicon wafer in a 30 wt% KOH solution at 60 °C; the thickness of the membrane after wet etching was 34 nm. Figure 5 shows a ZrSi_2_ pellicle composite with an area of 10 mm × 10 mm which was fabricated by depositing ZrSi_2_ thin films onto a SiN_x_ membrane via co-sputtering. The sputtering chamber was evacuated to a base pressure of less than 2 × 10^−7^ Torr. ZrSi_2_ thin films were deposited under pure Ar gas atmosphere at a pressure of 3 mTorr, and the substrate was heated to 500 °C. A Si sacrificial layer was deposited onto the ZrSi_2_ thin film to increase the EUV transmittance, and the SiN_x_ thin film was selectively etched at 150 °C using an 85 wt% H_3_PO_4_ solution. Finally, a ZrSi_2_-based pellicle was fabricated by selectively etching the Si sacrificial layer using KOH solution.

The composition ratio and crystal structure of the ZrSi_2_ thin film were analyzed using X-ray photoelectron spectroscopy (XPS) and X-ray diffraction (XRD), respectively. The thermal properties of the pellicle were evaluated using a heat load tester that measured the temperature of the pellicle heated by a 355 nm UV laser. To emulate an environment similar to EUV exposure, the chamber inside the heat load tester was maintained at a high vacuum, and a rotating slit was used to heat the pellicle in a 1:9 cycle. In addition, the Gaussian beam profile of the UV laser was adjusted to a top-hat profile using a diffractive optics element to ensure uniform laser incidence on the pellicle. A two-channel pyrometer with a measurable temperature range of 400–1500 °C and measurement accuracy of ±2% was used to measure the temperature of the pellicle [9,18].

(1) $$I=\frac{\alpha \cdot P}{D}$$

The absorbed heat flux density of the pellicle was calculated using Equation (1). Here, I is the absorbed heat flux density, D is the incident beam size, α is the absorptivity of the pellicle at a wavelength of 355 nm, and P is the laser power. Materials are generally cooled via convection, conduction, and radiation [9,19]. However, the high vacuum in the interior of the EUV scanner and the extremely low thickness of the EUV pellicle imply that cooling via convection and conduction can be ignored. Thus, the EUV pellicle is primarily cooled via radiation. The heat-transfer mechanism of the EUV pellicle is given by the following equation [18,20,21]:

(2) $$\frac{dT}{dt}=\frac{1}{c\cdot m}\left[\alpha \cdot P-\epsilon \cdot \sigma \cdot S\cdot \left(T^{4}-T_{s}^{4}\right)\right]$$

where c is the specific heat, m is the mass of the pellicle membrane, ϵ is the emissivity, σ is the Stefan–Boltzmann constant, T is the temperature of the pellicle membrane, and $T_{s}$ is the temperature of the surrounding air. The emissivity of the ZrSi_2_ pellicle composite was calculated from the results of the heat load test using Equation (2).

The EUV transmittance and reflectivity of the pellicle were measured using a coherent scattering microscope equipped with a 13.5 nm light source. The EUV transmittance was derived by comparing the number of photons reflected by the Mo/Si multilayers with and without the pellicle. The EUV reflectivity was calculated by comparing the number of photons reflected by the EUV pellicle when it was mounted on an absorber material where the EUV reflectivity converges to zero, with the number of photons reflected by the Mo/Si multilayers [22,23,24].

The mechanical properties of the EUV pellicle were evaluated via a bulge test, wherein the deflection of the membrane was measured as a function of the pressure difference on both sides of the membrane. In addition to the burst pressure, the residual stress, plane-strain modulus, and ultimate tensile strength (UTS) can be obtained from the bulge test [25,26,27]. A long rectangular membrane with an aspect ratio greater than 4:1 is required to obtain the mechanical properties; hence, a membrane with an area of 1.5 mm × 6 mm was used in this study. The strain and stress were calculated from the results of the bulge test using the following equations:

(3) $$\varepsilon =\frac{2h^{2}}{3a^{2}}+\varepsilon_{0}$$

(4) $$\sigma =\frac{pa^{2}}{2ht}$$

where ε is the strain, σ is the stress, h is the deflection at the center of the membrane, a is the half-width of the membrane, $\varepsilon_{0}$ is the initial strain, p is the applied gas pressure, and t is the thickness of the membrane. The y-intercept of the strain vs. stress curve obtained using the above equations represents the residual stress; the stress at the point where the membrane ruptures indicates the fracture strength, which is equivalent to the UTS of brittle materials.

## 4. Results and Discussion

**Figure 6:**
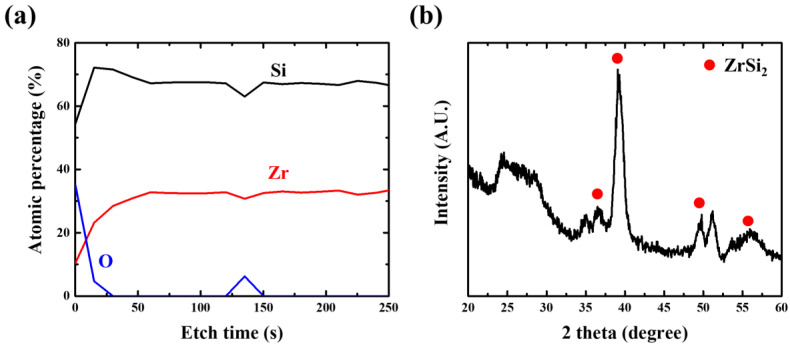
(**a**) XPS depth profile and (**b**) XRD diffraction patterns of ZrSi_2_ thin films.

Figure 6a shows the results of the XPS depth profile analysis of the ZrSi_2_ thin film. The Si/Zr ratio of the thin film was approximately 2. Moreover, the average oxygen content inside the thin film was less than 3 at% whereas the oxygen content on the surface of the thin film was higher due to oxidation. The XRD patterns shown in Figure 6b confirm the orthorhombic structure of the 40-nm-thick crystalline ZrSi_2_ thin film. The red dot represents the diffraction patterns of the ZrSi_2_ thin film with an orthorhombic structure. The broad halo pattern observed in the 2θ range of 24–30° corresponds to the diffraction pattern of the nanocrystalline phases of ZrSi_2_, SiO_2_, and ZrSiO_4_. Moreover, the peaks at 35° and 52° correspond to the diffraction patterns of Zr and Si wafer, respectively [28]. From these results, the ZrSi_2_ thin film was confirmed through composition and crystallinity analysis, and essential characteristics of the EUV pellicle were evaluated.

**Figure 7:**
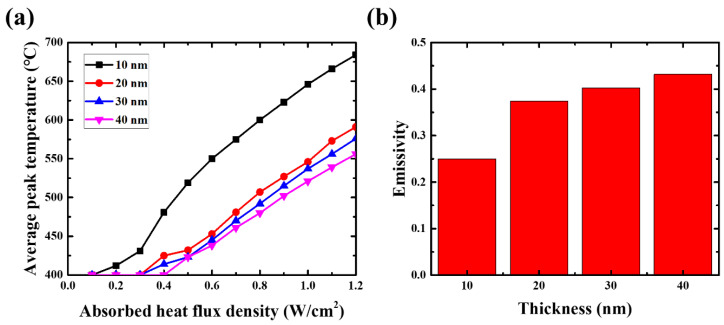
(**a**) Heat load test results of ZrSi_2_/SiN_x_ pellicle composite and (**b**) calculated emissivity of ZrSi_2_ thin films at various thicknesses.

A ZrSi_2_/SiN_x_ pellicle composite was fabricated by depositing 10-, 20-, 30-, and 40-nm-thick ZrSi_2_ thin films on a 34-nm-thick SiN_x_ membrane to examine the dependence of thermal properties on the thickness. Figure 7a shows the results of the heat load test: the pellicle composite with a thicker ZrSi_2_ thin film was heated to a lower temperature under identical absorbed heat flux density.

Kirchhoff’s law states that the emissivity of a material is equal to its absorptivity. The EUV pellicle is heated to temperatures in the range of hundreds of degrees Celsius, such that the emitted spectrum is mainly generated in the infrared (IR)-wavelength region during exposure. Therefore, the emissivity of the EUV pellicle can be calculated from the average absorptivity of a thin film in the IR-wavelength region [9,29]. A SiN_x_ thin film with a thickness of several tens of nanometers is transparent in the IR-wavelength region, which implies that its emissivity is close to zero. Therefore, the emissivity of the ZrSi_2_/SiN_x_ pellicle composite was assumed to be the same as that of the ZrSi_2_ thin film. Figure 7b shows the emissivity at different thicknesses of the ZrSi_2_ thin film. The emissivity was calculated from the results of the heat load test using Equation (2). The emissivity increased with an increase in the thickness of the ZrSi_2_ thin films. The calculated average emissivities of the 10-, 20-, 30-, and 40-nm-thick ZrSi_2_ thin films were 0.250, 0.374, 0.402, and 0.432, respectively. These values are similar to those of other materials used in EUV pellicle applications. Therefore, ZrSi_2_ is considered a suitable EUV pellicle material in terms of thermal properties.

The measured EUV transmittances of the ZrSi_2_/SiN_x_ pellicle composite were 82.8%, 74.9%, 73.6%, and 71.3%, respectively, at ZrSi_2_ thicknesses of 10, 20, 30, and 40 nm. However, an EUV transmittance of 90% or higher was necessary. Hence, a ZrSi_2_-based pellicle was fabricated using a Si sacrificial layer.

**Figure 8:**
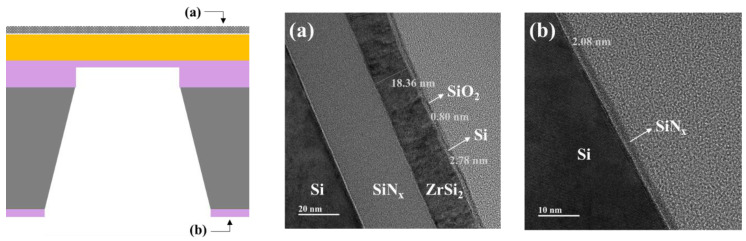
TEM cross-view images of the (**a**) top and (**b**) bottom of the frame area of the ZrSi_2_-based pellicle.

Figure 8 shows the TEM cross-sectional images of the ZrSi_2_-based pellicle. The structure and layer thickness of the membrane were estimated by analyzing the cross-section of the frame region. Figure 8a shows the top of the frame of the ZrSi_2_-based pellicle: an 18-nm-thick ZrSi_2_ layer and a 3-nm-thick Si layer including the surface oxide were observed. In addition, a 2-nm-thick SiN_x_ thin film was observed at the bottom of the frame, as shown in Figure 8b, which was expected to be the same thickness as that of the SiN_x_ layer of the ZrSi_2_-based pellicle. The measured EUV transmittance and reflectivity of the ZrSi_2_-based pellicle were 92.7% and 0.033%, respectively, and these values satisfy the EUV pellicle requirements.

**Figure 9:**
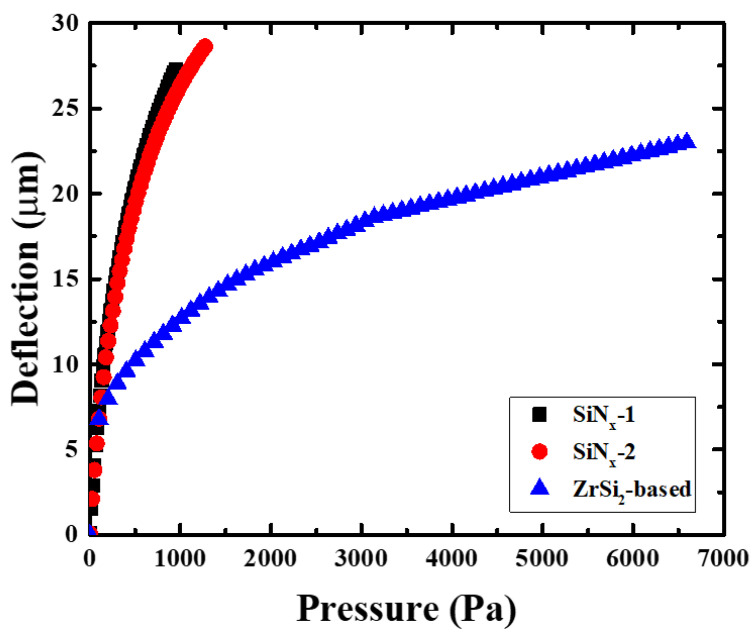
Results of the bulge test of the SiN_x_ membrane and ZrSi_2_-based pellicle.

The results of the bulge test of the SiN_x_ membrane and ZrSi_2_-based pellicle with similar EUV transmittances were compared to evaluate their mechanical durability as shown in Figure 9. The SiN_x_ membrane fractured at a pressure difference of 1116 Pa, whereas the ZrSi_2_-based pellicle fractured at an approximately 5.9 times higher pressure difference of 6592 Pa. Moreover, the deflection of the ZrSi_2_-based pellicle was lower than that of the SiN_x_ membrane at the same pressure difference. The strain and stress of the membrane were calculated from the bulge test results using Equations (3) and (4), respectively, and the residual stress and UTS were derived. The residual stress of the ZrSi_2_-based pellicle was −105 MPa. The UTS of the ZrSi_2_-based pellicle and SiN_x_ membrane were 3.5 and 0.5 GPa, respectively, and the superior mechanical properties of ZrSi_2_ were verified.

## 5. Conclusions

In this study, a range of optical constants that can be applied to an EUV pellicle material was presented using an optical simulation tool, and ZrSi_2_ was selected as a candidate for an EUV pellicle material considering its optical and thermomechanical properties. The composition ratio and crystal structure of the ZrSi_2_ thin film deposited via co-sputtering were confirmed by XPS and XRD analyses. Based on this, a ZrSi_2_/SiN_x_ pellicle composite was fabricated, and the relationship between the thickness and emissivity of the ZrSi_2_ thin film was investigated via a heat load test. To achieve a higher EUV transmittance, a ZrSi_2_-based pellicle was fabricated by introducing a Si sacrificial layer. The ZrSi_2_-based pellicle exhibited high EUV transmittance (>90%), low reflectivity (<0.04%), and high UTS (approximately 3.5 GPa), thereby satisfying the EUV pellicle requirements. These results demonstrate the excellent optical and thermomechanical properties of the nanoscale ZrSi_2_ thin film. Hence, ZrSi_2_ has potential applications as an EUV pellicle material that can withstand high-power EUV sources.

## Data Availability

The datasets generated and/or analyzed during the current study are available from the corresponding author upon reasonable request.
